# Comparing between steady-state excitonic transitions and ultrafast polaronic photoexcitations in layered perovskites: the role of electron–phonon interaction

**DOI:** 10.1515/nanoph-2023-0015

**Published:** 2023-04-20

**Authors:** Pingyuan Yan, Tao Li, Haoxiang Zhou, Shu Hu, Chenhong Xiang, Yang Zhang, Chengqiang Wang, Zihan Wu, Heng Li, Haibin Zhao, ChuanXiang Sheng

**Affiliations:** Department of Optical Science and Engineering, School of Information Science and Technology, Fudan University, Shanghai, 200433, China; School of Electronic and Optical Engineering, Nanjing University of Science and Technology, Nanjing, 210094, China

**Keywords:** 2D perovskite, electron-phonon interaction, excitonic transitions, optical properties, polarons

## Abstract

We have studied four 2D layered perovskites, including OA_2_PbI_4_ (RP phase), ODAPbI_4_ and BDAPbI_4_ (DJ phase), (GA)MAPbI_4_ (ACI phase), where OA is [(C_*m*_H_2*m*+1_)NH_3_](*m* = 8), ODA is [NH_3_(CH_2_)_*m*_NH_3_](*m* = 8), BDA is [NH_3_(CH_2_)_*m*_NH_3_](*m* = 4), and GA is [C(NH_2_)_3_]; RP, DJ, and ACI means Ruddlesden–Popper, Dion–Jacobson and alternating cations in the interlayer, respectively. The temperature dependence of absorption and photoluminescence (PL) spectra have been measured. From which the average phonon energy (electron-phonon interaction strength) is analyzed as around 34 (80), 47 (184), 50 (402), and 63 (758) with the unit of meV for OA_2_PbI_4_, ODAPbI_4,_ BDAPbI_4_, and (GA)MAPbI_4_, respectively. Larger phonon energy indicates the involvement of more phonons in organic spacer layer, with the corresponding stronger electron-phonon interaction. Furthermore, ultrafast transient absorption spectroscopy proves that, when the excitation photon energy is serval hundred meV higher than bandgap, the excitons still are the major photoexcitations in OA_2_PbI_4_, but polarons are major one in ODAPbI_4_, BDAPbI_4_, and (GA)MAPbI_4_ films, no matter the excitonic transitions dominate the absorption at their band edges. This work proves the organic spacers can regulate electron–phonon interaction then optoelectronic properties in 2D perovskites profoundly, which have implications toward future rational design for relevant devices.

## 1 Introduction

Two dimensional organic–inorganic hybrid halide perovskites (2DPKs) self-assemble into alternating organic and inorganic layers forming “multiple quantum wells” [1]. Recent interest was focused on applications in boosting efficiency and stability of both photovoltaic and light emitting diodes, which was initialed by the emerging of 3D hybrid perovskites in 2009 [2]. As an example of unconventional semiconductor [3], the 2DPKs present unique physical properties [4], including a “soft” structure [5], strong anisotropy [6], large binding energy of excitons [7], large Rashba splitting [8], and ferroelectric and nonlinear effects [9–11]. The layered 2D perovskites were furtherly classified according to the difference in organic spacer, including mainly Ruddlesden–Popper (RP) phase [12], Dion–Jacobson (DJ) phase [13], and alternating cations in the interlayer (ACI) phase et al. [14]. RP and DJ perovskites are by far the most studied, with formula of R_2_A_*n*−1_M_*n*_X_3*n*+1_ and R′A_*n*−1_M_*n*_X_3*n*+1_, respectively [15]. A is small cation CH_3_NH_3_^+^ (MA^+^), HC(NH_2_)_2_^+^ (FA^+^), Cs^+^, M is metal Pb^2+^ or Sn^2+^, and X is the halide Cl^−^, Br^−^, or I^−^ [16, 17]. R is an organic spacer cation that are largely held together through van der Waals interactions, and R′ is diamine compounds forming hydrogen bonds on both ends with the inorganic “quantum wells” [18]. ACI phase has formula of R″A_*n*_B_*n*_X_3*n*+1_. Small A cation exits not only in lead halide sheet, but also in the space layer with R″ cation, which Guanidinium (GA^+^) is the mostly reported for ACI perovskite so far adopting the layer-stacking characteristics of both DJ and RP structures [14, 19]. In summary, 2DPKs exhibit almost unlimited possibility, leading novel and exciting physical properties.

In the most 2D perovskite films, the inorganic layer plays a central role as the active (semiconducting) part of the system, particularly at the minimum of the energy bands. On the other hand, the organic layer is not just acting as barrier of quantum well. Recent studies reveal that strong and complex coupling between inorganic layer and the spacer cations strongly enhance the electron–phonon interaction, influence the electronic properties of the 2DPKs [7, 20]. However, fully understanding the importance of organic cations is still lacking, which is also partially due to that the most photo-physics studies had been focused on perovskite of RP phases, although the DJ and ACI phases have proved to be effective solar cell materials with long-term stability [21–23]. For example, layered ACI perovskite had demonstrated recently power conversion efficiency (PCE) of 22 % [24], which is among the highest values of 2DPKs solar cells.

In this work, we studied four 2D layered perovskites. To underline the importance of spacer cations, we only consider 2D-perovskites with *n* = 1, i.e., without containing 3D bulk-like slabs. The materials include OA_2_PbI_4_ (RP phase), ODAPbI_4_, BDAPbI_4_ (DJ phase), and (GA)MAPbI_4_ (ACI phase), where OA is [(C_*m*_H_2*m*+1_)NH_3_] (*m* = 8), ODA is [NH_3_(CH_2_)_*m*_NH_3_] (*m* = 8), BDA is [NH_3_(CH_2_)_*m*_NH_3_] (*m* = 4), and GA is [C(NH_2_)_3_]. The temperature dependence of absorption and photoluminescence (PL) spectra were measured. Because of enhanced stiffness in DJ and ACI perovskite compared to the RP one, intuitively smaller electron–phonon interaction is expected in them. However, on the contrary to this intuition, DJ phase and ACI phase present much stronger electron–phonon interaction compared to RP one in current work. The reason was attributed to the involvement of more phonon modes in organic spacer layer in DJ and ACI phases, which are suggested using Urbach analysis and PL spectral broadening as a function of the temperature. The ultrafast transient absorption spectroscopy measured at room temperature proves that, when the excitation energy of phonon is several hundred meV higher than bandgap, the excitons still are the major photoexcitations in OA_2_PbI_4_ of RP phase, but on the contrary, the polarons are dominant in ODAPbI_4_, BDAPbI_4_, and (GA)MAPbI_4_ films, no matter the excitons with binding energy all around 100 meV dominates the absorption at the band edge. Our work highlights the organic spacers can regulate electron–phonon interaction, which may manipulate optoelectronic properties in 2D perovskites profoundly.

## 2 Experimental

### 2.1 Starting materials

Octylammonium iodide (OAI, 99.5 %), 1,8-octanediammonium diiodide (ODAI_2_, 99.5 %), 1,4-butanediammonium diiodide (BDAI_2_, 99.5 %), guanidinium iodide (GAI, 99.5 %), methylammonium iodide (MAI, 99.5 %), and lead iodide (PbI_2_, 99.9 %) were purchased from Xi’an Polymer Light Technology Corp. N,N-Dimethylformamide (DMF, 99.9 %) and chlorobenzene (CB, 99.9 %) were purchased from Aladdin. All chemicals were used as received without further purified in this work.

### 2.2 Fabrication of films

For OA_2_PbI_4_ precursor solution was comprised of OAI and PbI_2_ (2: 1 M ratio) in 1 mL DMF solvents with a concentration of 0.4 mol/L (M). The ODAPbI_4_ and BDAPbI_4_ precursor solution was comprised of ODAI_2_ or BDAI_2_, and PbI_2_ (1: 1 M ratio) with a concentration of 0.8 M. The (GA)MAPbI_4_ precursor solution was comprised of GAI, MAI, and PbI_2_ (1: 1: 1 M ratio) in 1 mL DMF with 0.8 M. All solutions stirred overnight in glove box at 70 °C, finally cooled to room temperature (RT) and filter with a 0.22 µm PVDF syringe filter before used. The glass substrate was cleaned consecutively in detergent, acetone, isopropanol, and ethanol ultrasonic baths for 15 min, respectively. Then treated with O_3_ plasma for 30 min. All the films were spin-coated at 5000 rpm for 50 s onto the glass substrate, then annealed at 100 °C for 15 min (only (GA)MAPbI_4_ solution need 200 μL CB dropped onto the substrate at 10 s). Finally, all the films stored at N_2_ glovebox over 24 h for optical characterization.

### 2.3 Characterization and optical measurements

X-ray diffraction (XRD) patterns of the perovskite films were obtained using a Bruker AXS Dimension D8 X-ray System. The absorption spectrum was recorded by home-built system using a halogen lamp and spectrometer (PG2000, Ideaoptics). The PL spectra were obtained by home-built systems with an excitation of 447 nm continuous wave laser. Sample for absorption and PL was put in a liquid nitrogen-cooled cryostat in which temperature could vary from 80 K to 300 K. The transient absorption spectroscopy (TAS) measurements were performed using a Ti: Sapphire amplifier laser system (Spectra-Physics Lasers, 1 kHz, 100 fs). The 800 nm (1.55 eV) laser output was split into two beams. One beam was used to generate white light super-continuum for the probe in the spectral range from 1.7 eV to 2.8 eV for current work. The other beam’s second harmonic at 3.1 eV was used as the pump source, and the probe pulses were mechanically delayed with respect to the pump pulses using a translation stage. The pump and probe light overlapped on the sample placed in a vacuum cryostat. The transmitted probe was collected to record changes in transmission intensity (Δ*T*/*T*) induced by the pump light, where the negative Δ*T*/*T* is photoinduced absorption (PA) and the positive Δ*T*/*T* is photoinduced bleaching (PB) here.

## 3 Result and analysis

**Figure 1:**
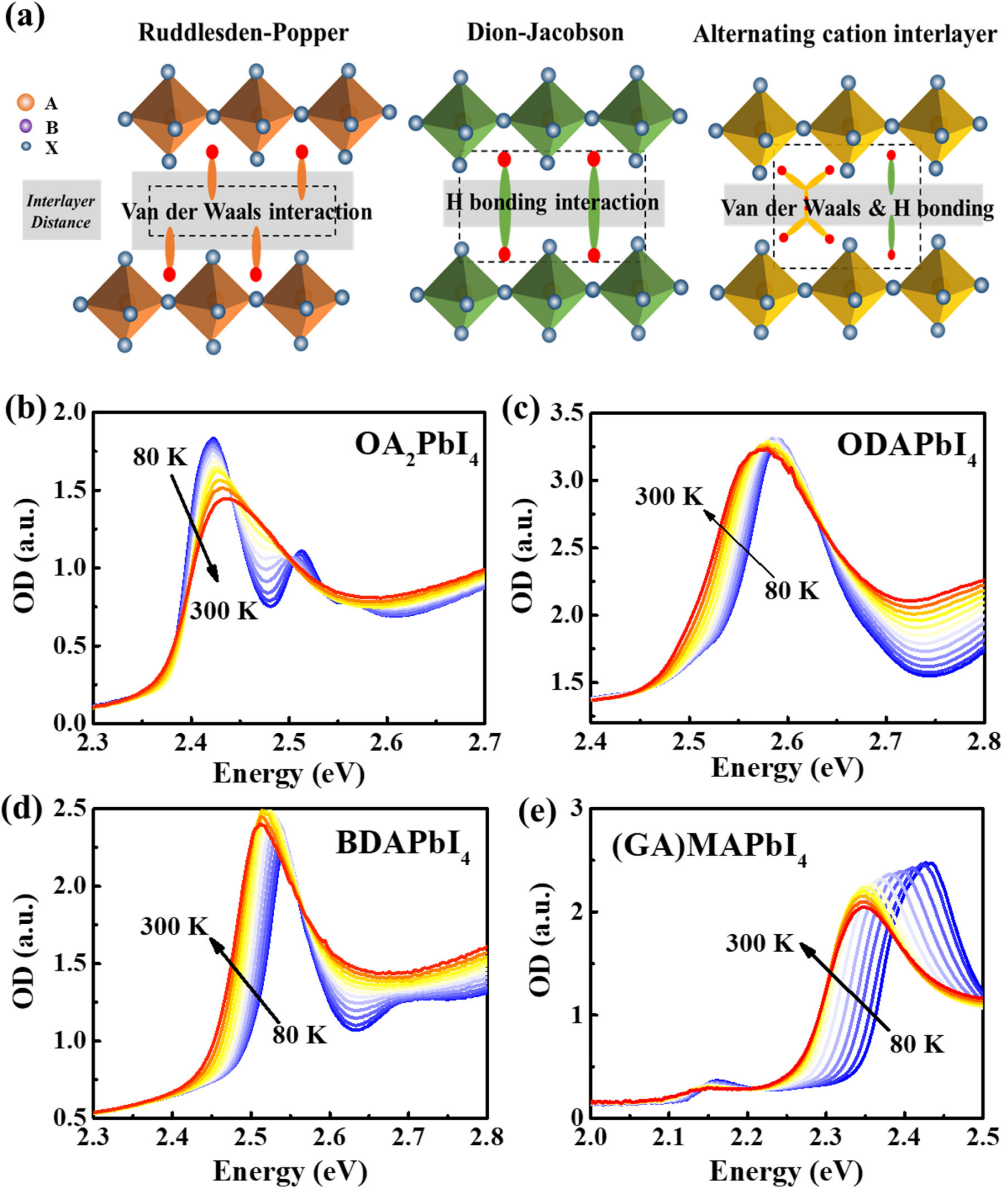
Schematic structures and Absorption spectra of layered perovskites of *n* = 1. (a) Schematic diagram of the structural comparison between RP type, DJ type, and ACI type. All interlayer cations interact with adjacent 2D perovskite slabs via hydrogen bonds which are symbolized using red point. DJ type eliminates the van der Walls interaction which exists in RP one. In ACI phase, two cations’ alternating arrangement retains mixed van der Waals interaction and H bonding [12–14]. The temperature dependence absorption spectra of 2D perovskites films, measured at various temperatures ranging from 80 K (blue line) and 300 K (red line): (b) OA_2_PbI_4_. (c) ODAPbI_4_. (d) BDAPbI_4_. (e) (GA)MAPbI_4_.

Figure 1a presents structures of RP-, DJ-, and ACI-phase 2D perovskite with *n* = 1, schematically. Figure 1(b–e) show absorption spectra at various temperatures ranging from 80 K and 300 K, for four 2D perovskites of *n* = 1, namely, OA_2_PbI_4_ (RP phase), ODAPbI_4_ (DJ phase), BDAPbI_4_ (DJ phase), and (GA)MAPbI_4_ (ACI phase), respectively. In Figure S1, we also included the absorption spectra of well-studied PEA_2_PbI_4_ (RP phase). All spectra contain prominent excitonic transitions that are consistent with a large exciton binding energy in the family of 2D hybrid perovskites with *n* = 1. XRD patterns are also included in Figure S2 of Supplementary Material, proving the good crystalline quality of the respective film.

In Figure 1, obviously, for OA_2_PbI_4_, as well as many other perovskites including 3D perovskite such as MAPbI_3_ and MAPbBr_3_ [25], the bandgap increases (blue-shifts) with increasing temperatures; on the other hand, the bandgap of two DJ perovskites BDAPbI_4_ and ODAPbI_4_ decrease (red-shift) with increasing temperatures. (GA)MAPbI_4_ also red-shifts obviously with increasing temperature below 200 K, but blue-shifts slightly above it. We note in (GA)MAPbI_4_ there is a weak absorption peak at 2.18 eV (570 nm) at 80 K, which may be due to the excitonic transition of *n* = 2 of (GA)MA_2_Pb_2_I_7_ [26], this is different with other *n* = 1 materials because of the existence of MAI in precursor solution.

In a semiconductor film, under constant pressure and a quasi-harmonic approximation, the temperature dependent bandgap of *E*_*g*_ can be simplified as [27, 28].

(1) $$E_{g}=E_{g0}+A_{TE}T+\sum_{q}A_{q}\left[2n_{q}+1\right]$$

where *E*_*g0*_ is the bandgap at 0 K, *A*_*TE*_ *T* describes the thermal expansion of the lattice, the third term on the right side of Eq. (1) corresponds to the contribution from the electron–phonon interaction; *A*_*TE*_ and *A*_*q*_ are parameters, *n*_*q*_ is the number of phonons with wave vector of *q*. *n*_*q*_ follows Bose–Einstein distribution of *1*/(exp(ℏ*w*_*q*_/*k*_*B*_ *T*)−1), where *ħw*_*q*_ is the energy of phonon with wave vector of *q*, and *k*_*B*_ is the Boltzmann constant.

In traditional semiconductors, such as silicon or GaAs, the *E*_*g*_ red-shifts with increasing temperature because of the electron–phonon interaction [29, 30]. For well-studied 3D perovskite such as MAPbI_3_ presents blue-shift of bandgap with increasing temperatures, mainly because the thermal expansion of the lattice reduces the overlap between Pb–6s and I–5p antibonding orbitals [31]. For 2D perovskites here, they all are composed of organic barrier layers and inorganic [PbI_6_]^4−^ octahedron layers forming multiple quantum wells, thus, the bottom of conduction band and top of valance band would be determined by Pb and I orbitals too. So naturally, the bandgap’s blueshift with increasing temperatures is expected in 2D perovskite, which is indeed observed in Figure 1a of OA_2_PbI_4_ and in literatures extensively [32, 33]. On the other hand, the red-shift bandgap with increasing temperatures should be ascribed to the second term in right hand of Eq. (1), i.e., the electron–phonon interaction.

**Figure 2:**
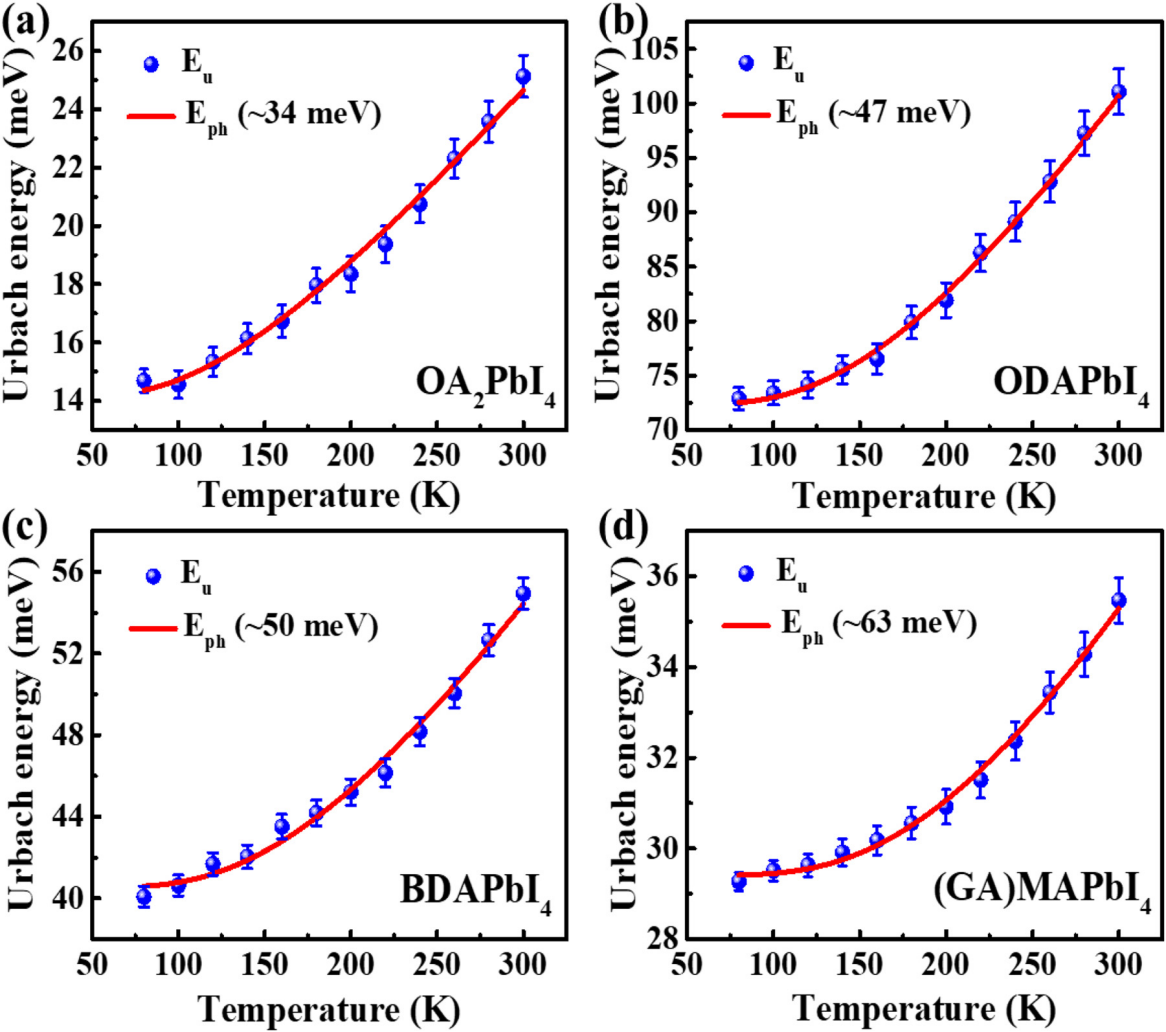
Urbach energy as a function of temperature of 2D perovskite films. The red curve is the fitting result using equation *E*_u_ ∝ *E*_ph_/tan*h*(*E*_ph_/2*k*_*B*_ *T*). (a) OA_2_PbI_4_. (b) ODAPbI_4_. (c) BDAPbI_4_. (d) (GA)MAPbI_4_.

To achieve information about phonons involving in optical processes, Urbach analysis, O.D. ∝ exp(*E*/*E*_u_), in which *E*_u_ is Urbach energy, was applied. In Figure 2, we present *E*_u_ as a function of temperature for OA_2_PbI_4_, ODAPbI_4_, BDAPbI_4_, and (GA)MAPbI_4_, respectively. The Urbach–Martienssen rule, in which *E*_u_ ∝ *E*_ph_/tan*h*(*E*_ph_/2*k*_*B*_ *T*) [34, 35] could be used to evaluate phonon energy that contributes to the optical transitions. Different materials present different *E*_ph_, which is 34 meV, 47 meV, 50 meV, and 63 meV, for OA_2_PbI_4_, ODAPbI_4_, BDAPbI_4_, and (GA)MAPbI_4_, as included in Figure 2. Here we noticed that the phonon energies from Urbach analysis are different in different materials and much larger than the phonon energy from Pb–I bonds, which is usually smaller than 16 meV [36, 37]. Therefore, the optical process could involve the multi-phonon modes from both organic and inorganic components in perovskite [38]. In 2DPKs, there may be three possible types of electron–phonon interaction: coupling to acoustic phonons, coupling to longitudinal optical phonon modes (so-called Fröhlich interaction) involving all atoms including in organic layer, and coupling to (out-of-plane) and (in-plane) optical phonons through deformation potentials [39–41]. For example, Thouin et al. reported the polaronic-related excitons in *n* = 1 2DPKs [40], in which case the phonons modes at frequencies below 2 THz (8.2 meV) related to a combination of perovskite octahedral twist and tilt modes were reported [42, 43]. Thus, multiple phonon scattering in optical processes is almost inevitable [39, 41, 42].

**Figure 3:**
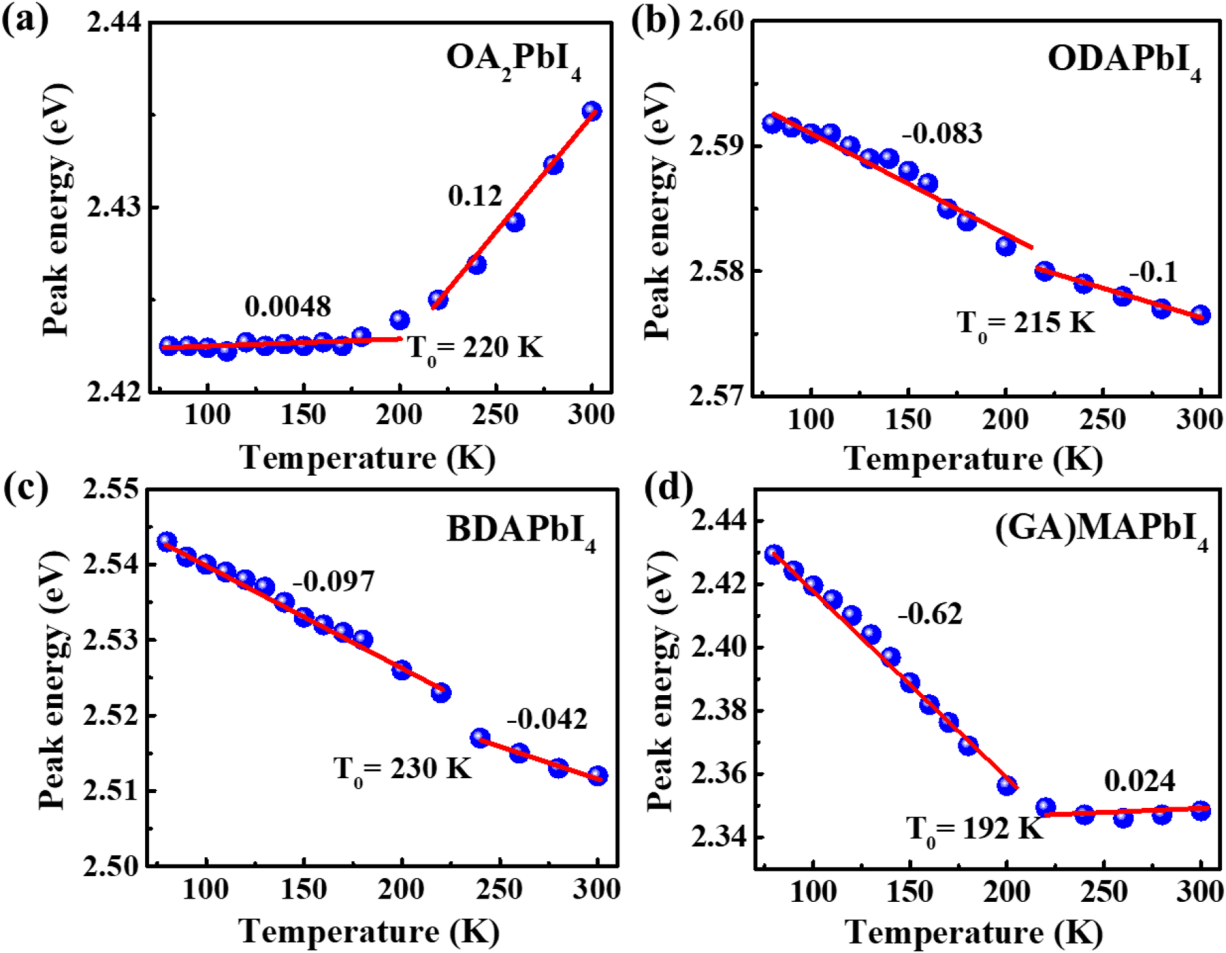
The exciton peak energy of the absorption spectrum in 2D perovskite, plotted as a function of the temperature (symbols represent data; the solid line is the linear fitting). (a) OA_2_PbI_4_. (b) ODAPbI_4_. (c) BDAPbI_4_. (d) (GA)MAPbI_4_.

The summation in Eq. (1) requires the involvement of all possible phonon modes within the Brillouin zone, this is actually impossible for 2D perovskite; thus one oscillator model was extensively used [44–46]. However, as shown in Figure 3 of peak positions of absorption, it cannot be described using one oscillator approximation. (Please see Figure S3 and its discussion).

On the other hand, Eq. (1) can approximately equal *A*_*TE*_ *T + S*_*EP*_ *T*, where *S*_*EP*_ relates to the electron phonon coupling strength, at relatively high temperature compared to the phonon energies [47, 48]; This is also consistent with the almost linear relation between peak energy and temperature shown in Figure 3, if the data was separated for two parts.

Since the separating of contributions from bandgap’s modification and electron–phonon interaction is impossible, only the value of (*A*_*TE*_ + *S*_*EP*_) was included in Figure 3, with unit of meV/K. (Here we should point out that a much “better” fitting using single oscillator approximation can be achieved numerically, if two separated parts in Figure 3 were fitted separately; however, because the linear nature of the curve, the fitting is not reliable, i.e., unrealistic large *A*_*TE*_ and almost *zero* electron–phonon interaction would be the results). Nice fitting in Figure 2 proves the electron–phonon interaction doesn’t change dramatically for respective material within temperature range, thus although the values of *S*_*EP*_ would be very different among materials discussed in current work, they should not be a function of temperature for each of films; therefore, we may conclude that the *A*_*TE*_ shows the dramatic change for OA_2_PbI_4_, ODAPbI_4_, BDAPbI_4_, and (GA)MAPbI_4_, respectively. Since the *A*_*TE*_ is related to the overlapping degrees of atomic orbitals [31], so we believed that the *T*_0_ shown in Figure 3 are related to the phase change temperature, as also suggested by Ref [45] measured in CsPbBr_3_. For instance, the *T*_0_ of OA_2_PbI_4_ in Figure 3a is 220 K, which is consistent with the reported value of 235 K [49, 50]. The *T*_0_ of BDAPbI_4_ in Figure 3c is 230 K, the reported phase change temperature is about ∼250 K for single crystal of BDAPbI_4_ [51]. Also, as shown in Figure S4, there is no obvious change of slope for PEA_2_PbI_4_, this is consistent with no phase change reported for PEA_2_PbI_4_ within the temperature ranging from 80 K to 300 K. On the other hand, although there is no report about phase change behavior for (GA)MAPbI_4_, it can suggest that happens around 190 K. Although detailed discussion between *A*_*TE*_ and the phase change is out of the scope of current work, we may like to suggest the following parameters could be helpful in understanding the topic: The angle of Pb–I–Pb bonds which could be different before and after phase change; The involvement of organic spacer in 2D perovskite since the wave-function of Pb and I orbitals may extend into organic barriers; The interaction between adjacent perovskite slabs, particularly for DJ and ACI phases in which the interlayer distances are relatively smaller and interaction are stronger [52–54].

**Figure 4:**
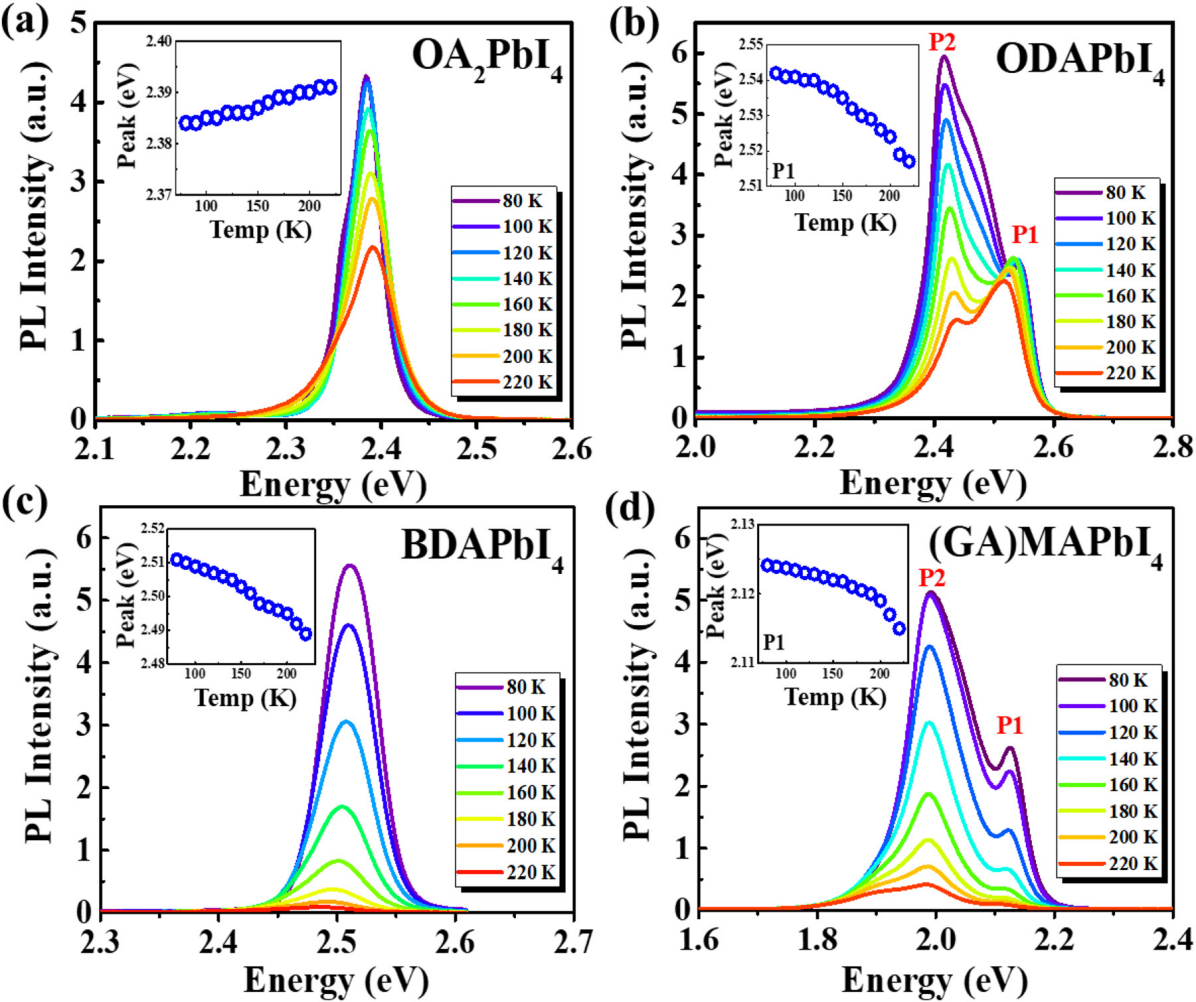
Temperature-dependent PL spectra of 2D perovskite films, measured various temperatures ranging from 80 K (blue line) and 220 K (red line). (Inset) Energy of the exciton peak in the PL spectra. (a) OA_2_PbI_4_. (b) ODAPbI_4_. (c) BDAPbI_4_. (d) (GA)MAPbI_4_.

Furthermore, another way to investigate the effects of electron-phonon interaction in semiconductors is to analyze PL spectra. In Figure 4, temperature dependent PL spectra of OA_2_PbI_4_, ODAPbI_4_, BDAPbI_4_, and (GA)MAPbI_4_ were presented up to 230 K, respectively. For ODAPbI_4_, and (GA)MAPbI_4_, there are mainly two peaks, whereas P1 is at higher energy, which may be due to the free excitons, and P2 is at lower energy which could be due to the self-trapped excitons [55] or trap states [56].

In Supplementary Material, normalized PL spectra up to 300 K is shown in Figure S5, obviously for ODAPbI_4_ and (GA)MAPbI_4_, the energy position of P1 is not reliable anymore, thus in the respective inset, the energy position of P1 was summarized up to 220 K. On the other hand, although it is not exactly same, the change of peak position in PL for OA_2_PbI_4_, ODAPbI_4_, BDAPbI_4_ are similar to that in absorption shown in Figure 3. (The peak positions of PL and absorption are compared directly in Figure S6). But not for the (GA)MAPbI_4_, unlike the absorption, the peak position of PL keeps almost constant below 200 K, and redshifts obviously above 200 K. Thus, we may conclude that the PL in (GA)MAPbI_4_ results from different species that are responsible to absorption. The direct comparison between PL and absorption at 80 K in Figure S7 may prove that the P1 in Figure 4d for (GA)MAPbI_4_ is due to the *n* = 2 of (GA)MA_2_Pb_2_I_7_ [26].

**Figure 5:**
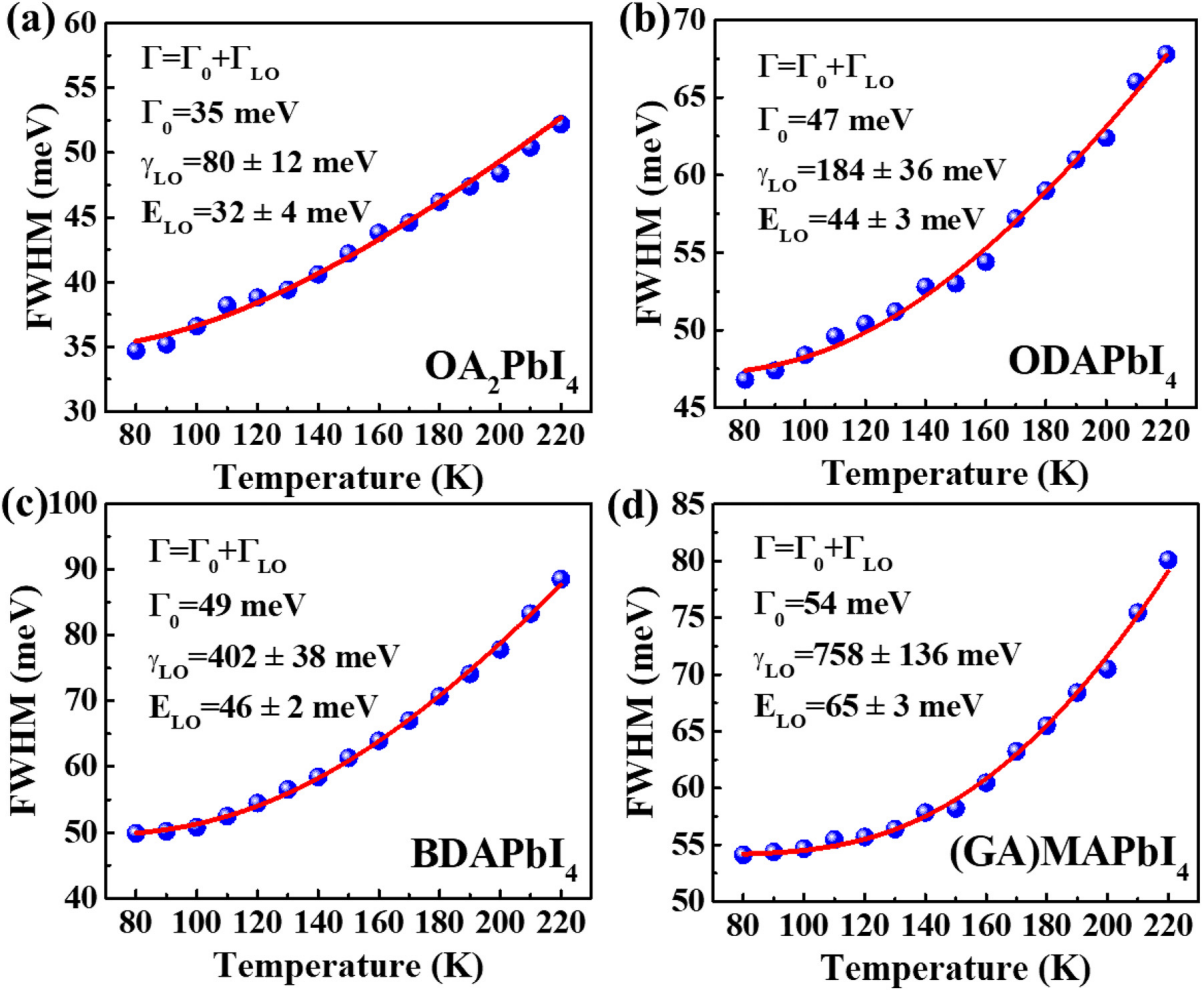
The full width at half maximum (FWHM) of the PL spectra plotted as a function of temperature (symbols represent data; the red line is the fitting curve using Eq. (2).) (a) OA_2_PbI_4_. (b) ODAPbI_4_. (c) BDAPbI_4_. (d) (GA)MAPbI_4_.

The PL width broadening with increasing temperatures has been used to extract information about electron-phonon interaction in semiconductors [57]. Figure 5 shows the temperature dependence of the full width at half maximum (FWHM) of peak P1 after respective fitting (not shown) from the PL spectra in Figure 4, particularly, the FWHM of P1 peak for ODAPbI_4_ and GAMAPbI_4_ were singled out by multiple-peak Gaussian fitting. The Following equation is to fit the FWHM as a function of temperature:

(2) $$\Gamma_{0}\left(T\right)=\Gamma_{0}+\Gamma_{LO}+\gamma_{LO}/\left(e^{E_{LO}/K_{B}T}-1\right)$$

where Γ_0_ is inhomogeneous broadening without temperature dependence, the second term *γ*_LO_ is the electron-phonon coupling strength for Fröhlich scattering, *E*_*LO*_ is energy of the *LO* phonon, and *k*_*B*_ is the Boltzmann constant. Here, we ignore the contribution of acoustic phonons and impurities for broadening PL, because the impurities scattering will show saturation of broadening at high temperature, and acoustic phonon scattering, which could be more important at low temperature, will present linearly broadening [37]. The fitting parameters are included in Figure 5 and summarized in Table 1. The similar values of phonon energy from Urbach analysis and PL broadening may indicate that the similar group of phonons is responsible to absorption and PL here.

**Table 1:** The fitting parameters of phonon energy from Urbach analysis and FWHM of PL. (^a^from Urbach analysis, ^b^from FWHM of PL spectra).

Sample	Layer distance(nm)	Phonon energy^a^(meV)	*A*_*TE*_ + *S*_*TE*_(<*T*_0_)(meV/K)	*A*_*TE*_ + *S*_*TE*_(>*T*_0_)(meV/K)	*T*_0_ (K)	Phonon energy^b^ (meV)	*γ*_LO_ (meV)
OA_2_PbI_4_	1.90 [49, 50]	34	0.0048	0.12	220	32	80
ODAPbI_4_	0.83 [32, 58]	47	−0.083	−0.10	215	44	184
BDAPbI_4_	0.43 [27, 58]	50	−0.097	−0.042	230	46	402
(GA)MAPbI_4_	0.318 [14]	68	−0.62	0.024	192	65	758
PEA_2_PbI_4_	1.6 [59, 60]	30 [61]	0.1 [61]	0.1 [61]	*–*	29 [61]	70 [61]

Since the value of *A*_*TE*_ is not available, the precise relation between *S*_*ET*_ (phonon–electron coupling strength from Urbach analysis) and *γ*_LO_ (phonon–electron coupling strength from PL analysis) cannot be achieved. However, the positive correlation is obvious, namely, more red-shift of the absorption peak in absorption spectra, stronger electron–phonon interaction exits in PL processes in general.

**Figure 6:**
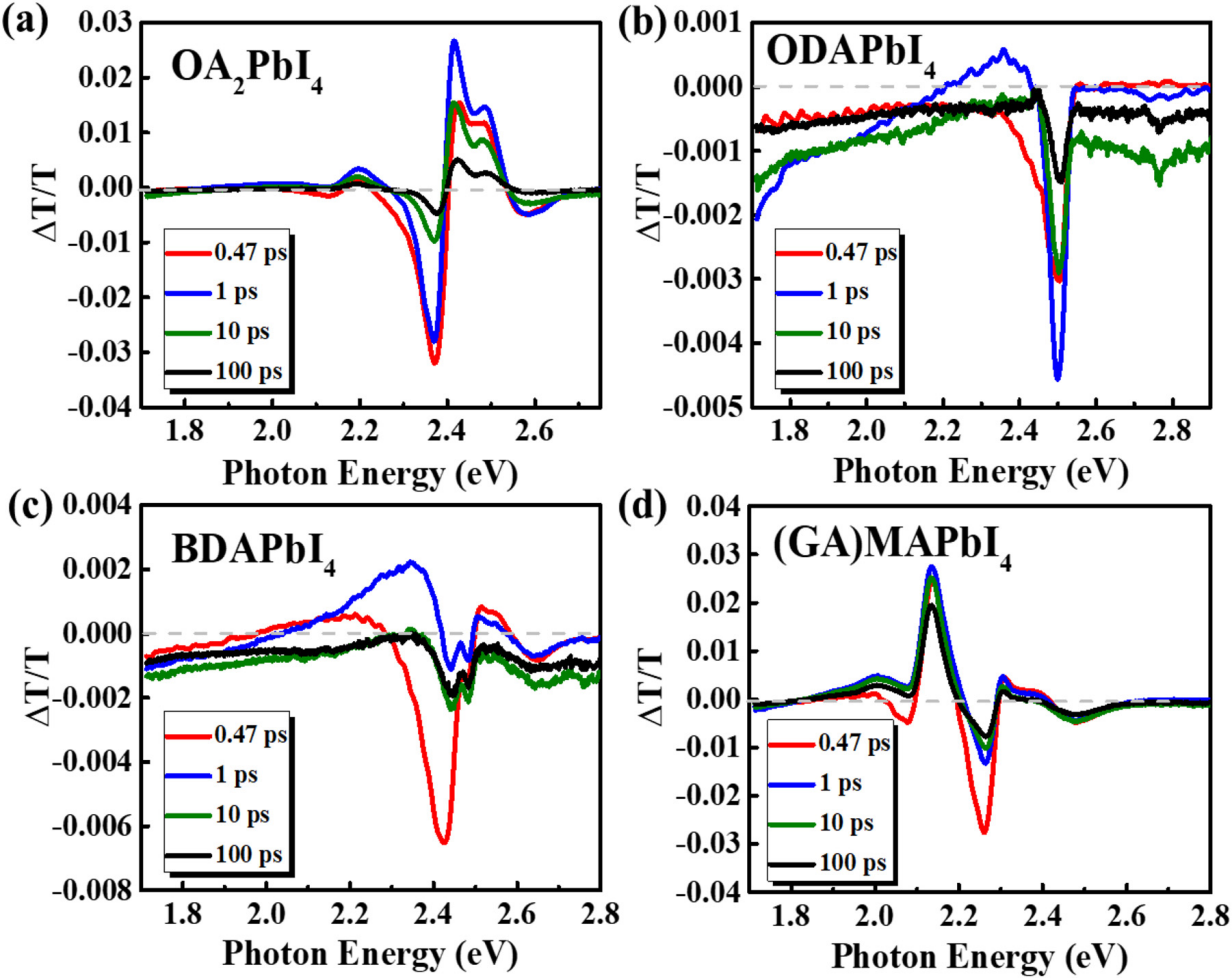
Ultrafast transient absorption spectra of four films at various delay times excited at 3.1 eV (400 nm). (a) OA_2_PbI_4_. (b) ODAPbI_4_. (c) BDAPbI_4_. (d) (GA)MAPbI_4_.

The results for strong electron phonon interaction may be the generation of polarons as photo-generated carriers directly [62]. In Figure 6, we summarized the transient absorption spectra (TAS) of four films at various delay times excited at 3.1 eV (400 nm). For OA_2_PbI_4_ of RP phase, although the delayed maximum of photo-bleaching (PB) peak may suggest the slow thermalization happens in OA_2_PbI_4_ since the pump photon energy is 0.65 eV higher than exitonic transition (2.44 eV in Figure 1b), the photoinduced features around band edge can be plausibly explained by a combination of “band filling” due to the excitonic transition around 2.45 eV [63], photoinduced symmetry-breaking process (such as spatially inhomogeneous strain or photoinduced electric field [64, 65]), and transient broadening [66]. From the dominant exciton PB band in the Figure 6a for OA_2_PbI_4_, we conclude that the exciton is formed after photon’s absorption within 1 ps, and remains as major photoexcitations within the time domain shown in Figure 6a.

However, for DJ perovskite of ODAPbI_4_, the PB band which should be around 2.58 eV totally disappears. This is also proved as the dynamics shown in Figure S8a, in which there is no any sign of PB signal for band-filling effect at the probe energy of 2.58 eV (the excitonic peak in absorption spectrum in Figure 1c). On the other hand, a strong PA signal appears at 1.73 eV, which is far below the band edge, and obviously it is rising with increasing wavelengths, which could be ascribed to the photogenerated polaron’ absorption [67]. Thus we conclude that with the excitation at 3.1 eV, which is 500 meV higher than excitonic transition, the exciton may not be formed, on the other hand, the polaron could be formed directly under this non-resonant excitation, if the difference between photon energy and bandgap is much larger than the binding energy of excitons [68]. Since the binding of exciton in ODAPbI_4_ is estimated to be 138 meV using 2D Elliott model (see Figure S9 and its discussion), which is similar with the binding energy of OA_2_PbI_4_ (101 meV), thus the formation of polaron directly could be due to the stronger electron-phonon interaction in ODAPbI_4_ of DJ phase.

For BDAPbI_4_, at the time of ∼0 ps, the PB band for band filling of absorption peak shown in Figure 1d is really weak and changes to PA structure swiftly, at the same time, the PA signal shown in Figure S8b shows a rising process of PA signal at 1.7 eV, suggesting the formation and dissociation of excitons, which is consistent with the increase of PA signal at 1.7 eV (polaron). Considering the large signal at around *t* = ∼0 ps, we may conclude both polaron and exciton are photogenerated simultaneously in BDAPbI_4_ within the time resolution.

For (GA)MAPbI_4_, the excitonic peak of *n* = 1 is at 2.34 eV. However, there is additional band filling at 2.15 eV, which may be due to the *n* = 2 materials in film, this is also consistent with the PL spectra. The simultaneous generated PA signal at 1.72 eV (Figure S8c), proves the photogenerated polaron at the beginning, which is consistent with the large electron–phonon interaction in film and relatively small *E*_b_ of binding energy (107 meV) achieved from Elliott model too (Figure S9e).

In general, except for RP perovskite of OA_2_PbI_4_, the other three films present the almost disappearance of exciton band in transient absorption spectroscopy even at *t* = ∼0 ps, with obvious photoinduced absorption which could be due to the formation of polarons.

Although the detailed dynamics of polaron’s photo-generation and formation of excitons as well as its dissociation is out the scope of current work, we may still conclude that the electron–phonon interaction results in the direct generation of photo-carriers in film, thus proving that the electron–phonon interaction could play dominant role in optoelectronic properties in 2D perovskite if it is strong.

## 4 Discussions and conclusions

Normally, the extrema point in conduction band and valence band of semiconductor are determining the optical and transport properties of these materials. Thus, although the large binding energy of exciton (usually around or larger than 100 meV for *n* = 1 2D perovskite at room temperature) is mainly due to the dielectric confinement effect caused by organic spacer, the organic cations and organic layers are still thought to constitute no primary role in the wave functions close to the band edges, but indirectly influence them by deforming the lattice and tuning the interlayer distance of the inorganic planes. However, the in-depth studies already revealed the important roles of the spacer cations in determining the photophysical properties, partially by electron–phonon interaction.

For RP type 2D perovskite, the longer organic spacer normally brings more mechanically ‘soft’ 2D perovskite; this will significantly enhance the electron–phonon interaction [69–72]. For example, dodecylammonium (DA) lead iodide, (C_12_H_28_N)_2_PbI_4_, result in red-shift of bandgap with increasing temperature, compared to hexylammonium (HA) lead iodide, (C_6_H_16_N)_2_PbI_4_ [53]. On the other hand, in the DJ phase, the presence of a single spacer molecule was thought to enhance the lattice stiffness, thus weakening electron–phonon coupling. However, as shown in current work, both DJ 2D perovskites, although with smaller layer distance, present stronger electron–phonon interaction. We believe that the reason could be due to the noncovalent interlayer halogen interactions promote increased electronic coupling between the layers, in other word, the wave-function of Pb and I orbitals would be more delocalized on the direction perpendicular of the quantum well, thus the phonons in organic layers will contribute more to electronic states in the bottom of quantum wells. Another consequence of interaction enhancement would be more distortion of Pb–I–Pb bonds, which then reduces the overlap between Pb–6s and I–5p antibonding orbitals, this will cause lesser blue-shift of band gap with increasing temperatures too [73, 74]. Furthermore, between the two 2D DJ perovskites in current work, the longer organic spacer also did not bring “stronger” electron–phonon interaction too, which strengths the argument in current works.

The more fundamental consequence of the large electron–phonon interaction may be the formation of so-called exciton–polarons caused by thermally activated lattice distortion while being excited off-resonantly [71]. This lattice distortions surrounding electron and hole, which opposite to each other would screen Coulomb interaction between them, thus lowering binding energy of excitons compared to the value extracted from steady-state UV–vis absorption spectra. Thus when a photon is absorbed in DJ and ACI of *n* = 1 perovskites in current work, although the binding energy of exciton is around 100 meV from absorption spectra, the charged polarons but not neutral excitons could determine the optoelectronic properties [75].

In conclusion, we measured the temperature dependence of absorption and PL spectra of four layered perovskite with *n* = 1, namely, OA_2_PbI_4_ (RP phase), ODAPbI_4_ (DJ phase), BDAPbI_4_ (DJ phase), and (GA)MAPbI_4_ (ACI phase). The presence of a single spacer molecule in DJ phase perovskite will enhance the lattice stiffness, and ACI of *n* = 1 has shortest distance between the inorganic layer; however, counterintuitively, DJ phase and ACI phase presents much larger electron-phonon interaction strength compared to RP one. The reason is ascribed to the more phonon modes from organic spacer would be involved in the optoelectronic processes in inorganic quantum well layer. The further ultrafast transient absorption spectroscopy measurement proves that, with the excitation energy off-resonantly, the excitons still are the major photoexcitations in OA_2_PbI_4_ of RP phase because of its relative small electron–phonon interaction strength, but on the contrary, the polarons are dominant in ODAPbI_4_ and BDAPbI_4_, (GA)MAPbI_4_ film, being consistent with their strong electron–phonon interaction. Our work provides fundamental insights into photophysics of the 2D perovskite with direct implication for optoelectronics.

## Supplementary Material

Supplementary Material Details
