# Supplementary material for: Comparing between steady-state excitonic transitions and ultrafast polaronic photoexcitations in layered perovskites: the role of electron–phonon interaction
Source: Nanophotonics. 2023 Apr 20;12(11):1965–77. doi: 10.1515/nanoph-2023-0015 (PMC11501284; doi:10.1515/nanoph-2023-0015)
Supplement: Supplementary file 1 — Supplementary Material Details [file j_nanoph-2023-0015_suppl_001.doc]

**Supplemental Information**

**Comparing between steady-state excitonic transitions and ultrafast polaronic photoexcitations in layered perovskites: the role of electron-phonon interaction**

Pingyuan Yan ^1, 2, #^, Tao Li ^1, #^, Haoxiang Zhou ^1^, Shu Hu ^2^, Chenhong Xiang ^2^, Yang Zhang ^2^, Chengqiang Wang ^2^, Zihan Wu ^2^, Heng Li ^2^, Haibin Zhao ^1^,*, ChuanXiang Sheng ^1, 2^,*

^1^ Department of Optical Science and Engineering, School of Information Science and Technology, Fudan University, Shanghai, 200433, China

^2^ School of Electronic and Optical Engineering, Nanjing University of Science and Technology, Nanjing, 210094, China

^#^ These authors contributed equally.

*Corresponding author. E-mail: [cxsheng@fudan.edu.cn](mailto:cxsheng@fudan.edu.cn) (C.-X.S.); [hbzhao@fudan.edu.cn](mailto:hbzhao@fudan.edu.cn) (H.-B.Z.)


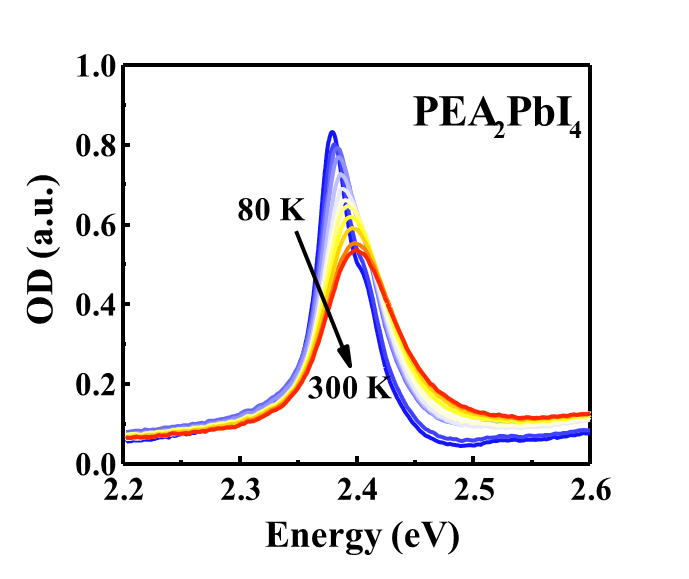


**Figure S1.** The temperature dependence absorption spectra of PEA_2_PbI_4_ films, measured at various temperatures ranging from 80 K (blue line) and 300 K (red line).


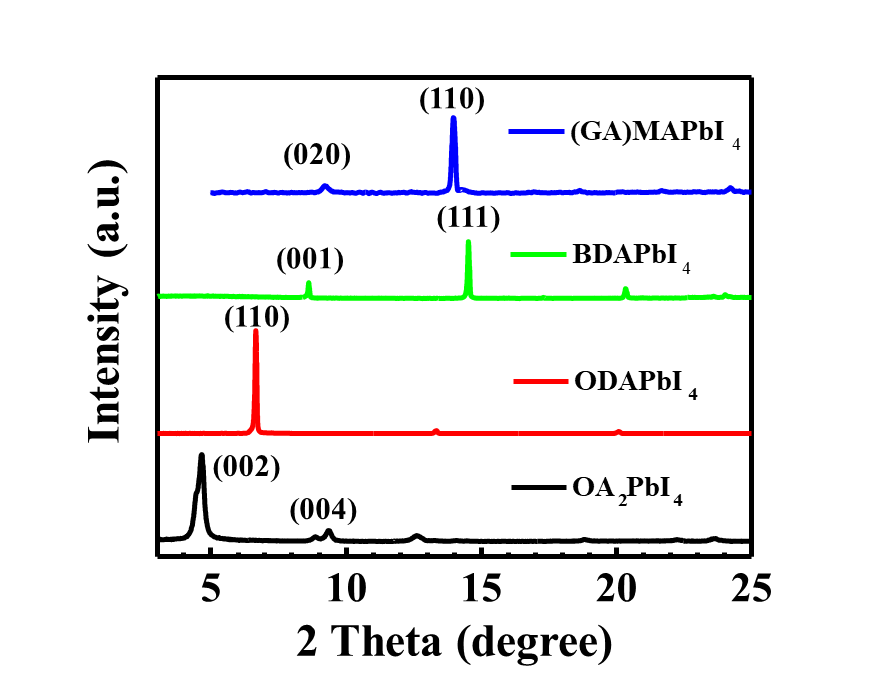


**Figure S2.** X-ray diffraction (XRD) patterns of 2D perovskite films. (The main diffraction peaks of OA_2_PbI_4_ was correspond to (002) crystal planes [1], and main peaks of ODAPbI_4_, BDAPbI_4_, (GA)MAPbI_4_ were assigned to (110) plane [2], (111) plane [3], (110) plane [4], respectively.)

**
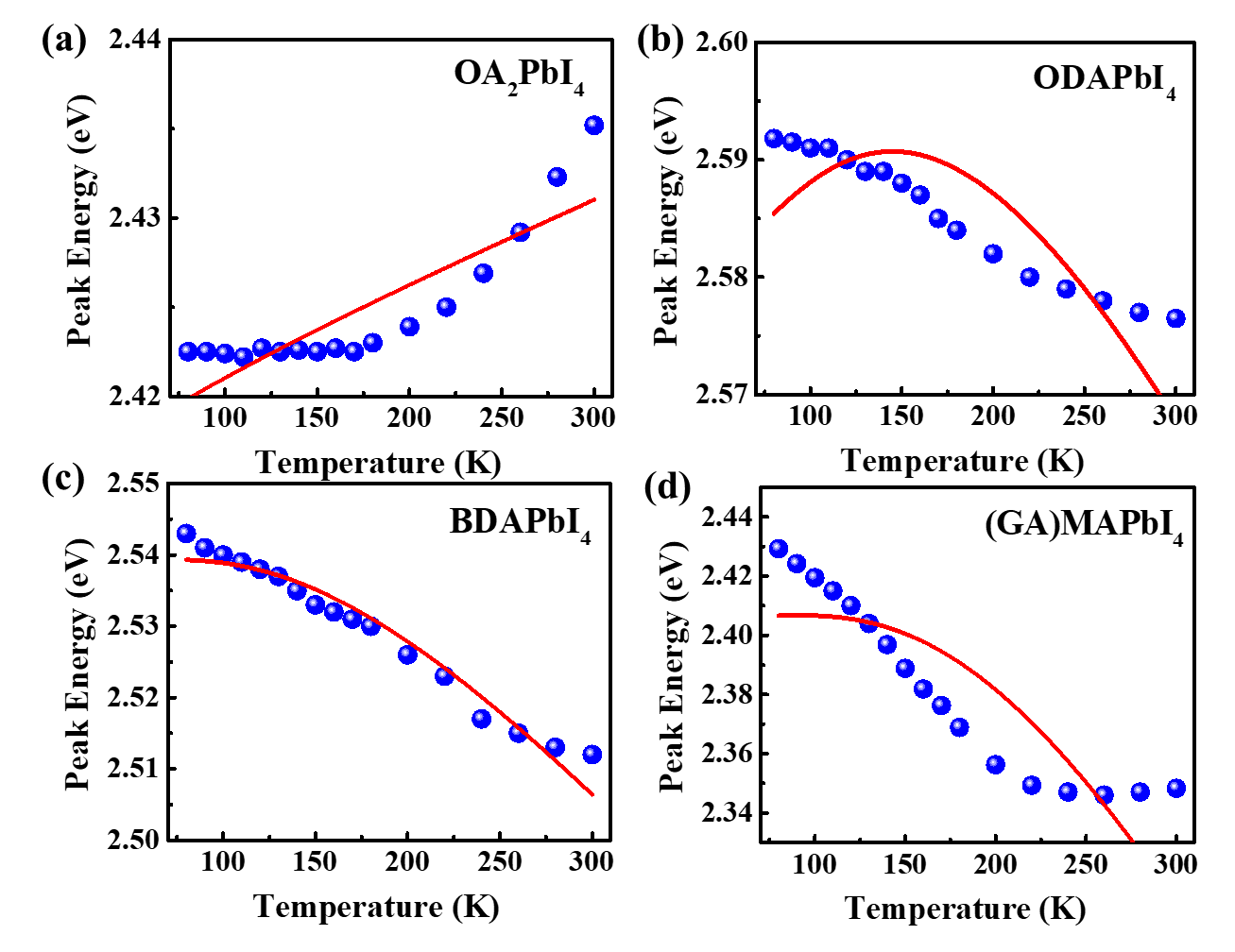
**

**Figure S3.** The exciton peak energy of the absorption spectrum in 2D perovskite, plotted as a function of the temperature (symbols represent data; the solid line is the fitting with the one-oscillator model shown in following). (a) OA_2_PbI_4_. (b) ODAPbI_4_. (c) BDAPbI_4_. (d) (GA)MAPbI_4_.

The temperature evolution of the band gap *E_g_*(T) in one-oscillator model is [5]:

Eq. S1

where *E*_0_ is the unrenormalized bandgap, *A_TE_* and *A_EP_* are the weight of the thermal expansion and electron-phonon interaction, respectively, and ℏω is the averaged optical phonon energy. Obviously, the Eq. S1 can not be used to fit the data shown in Figure S3 successfully.

**
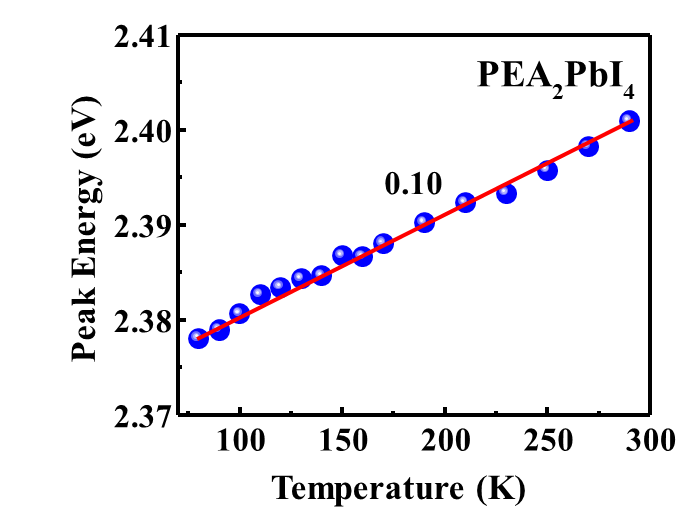
**

**Figure S4.** Energy of the exciton peak in the PEA_2_PbI_4_ spectra. (symbols represent data; the solid line is the linear fitting with a coefficient of 0.1 meV/K.).

**
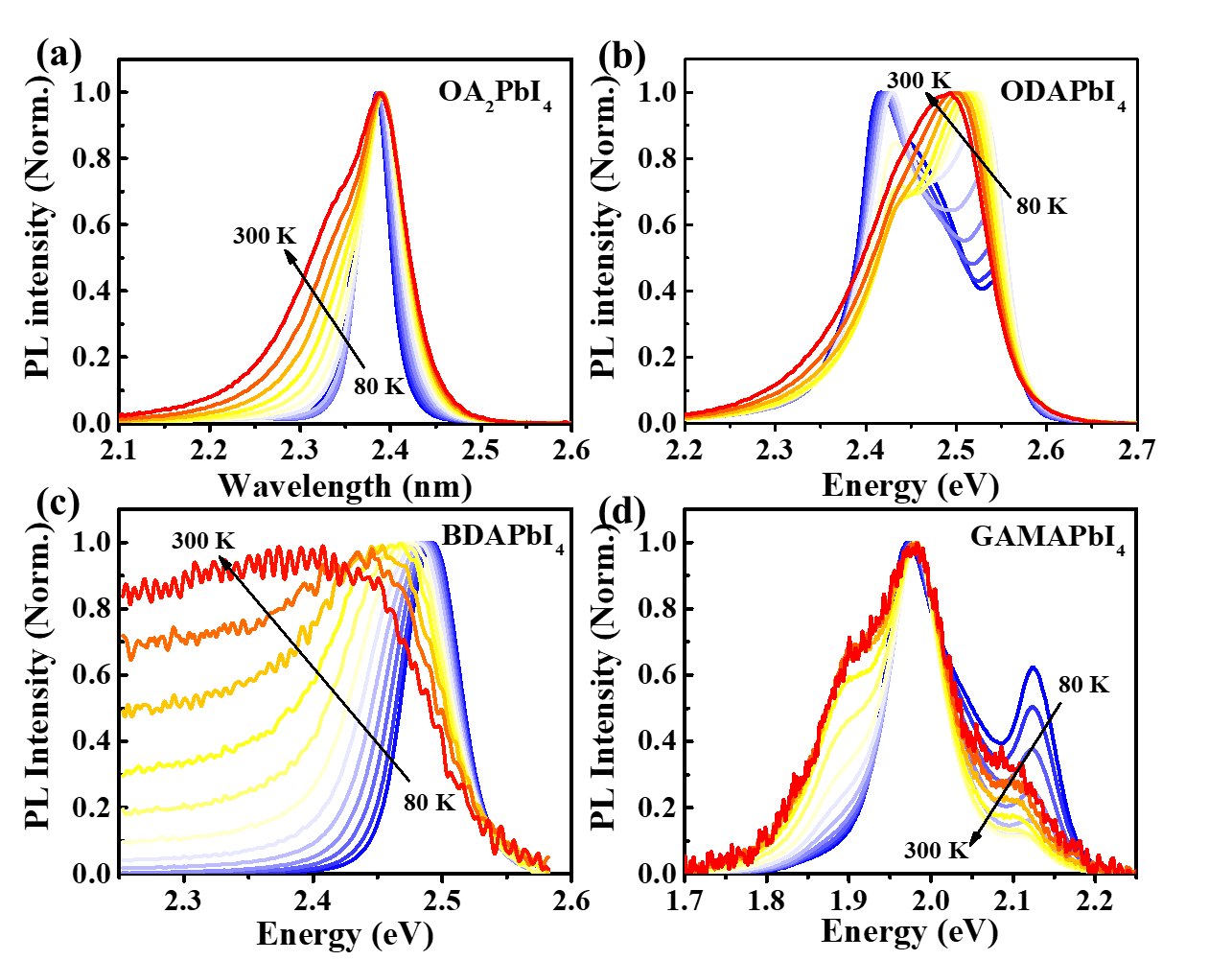
**

**Figure S5.** Temperature-dependent normalized PL spectra of 2D perovskite films, measured at various temperatures ranging from 80 K (blue line) and 300 K (red line). (a) OA_2_PbI_4_. (b) ODAPbI_4_. (c) BDAPbI_4_. (d) (GA)MAPbI_4_.

**
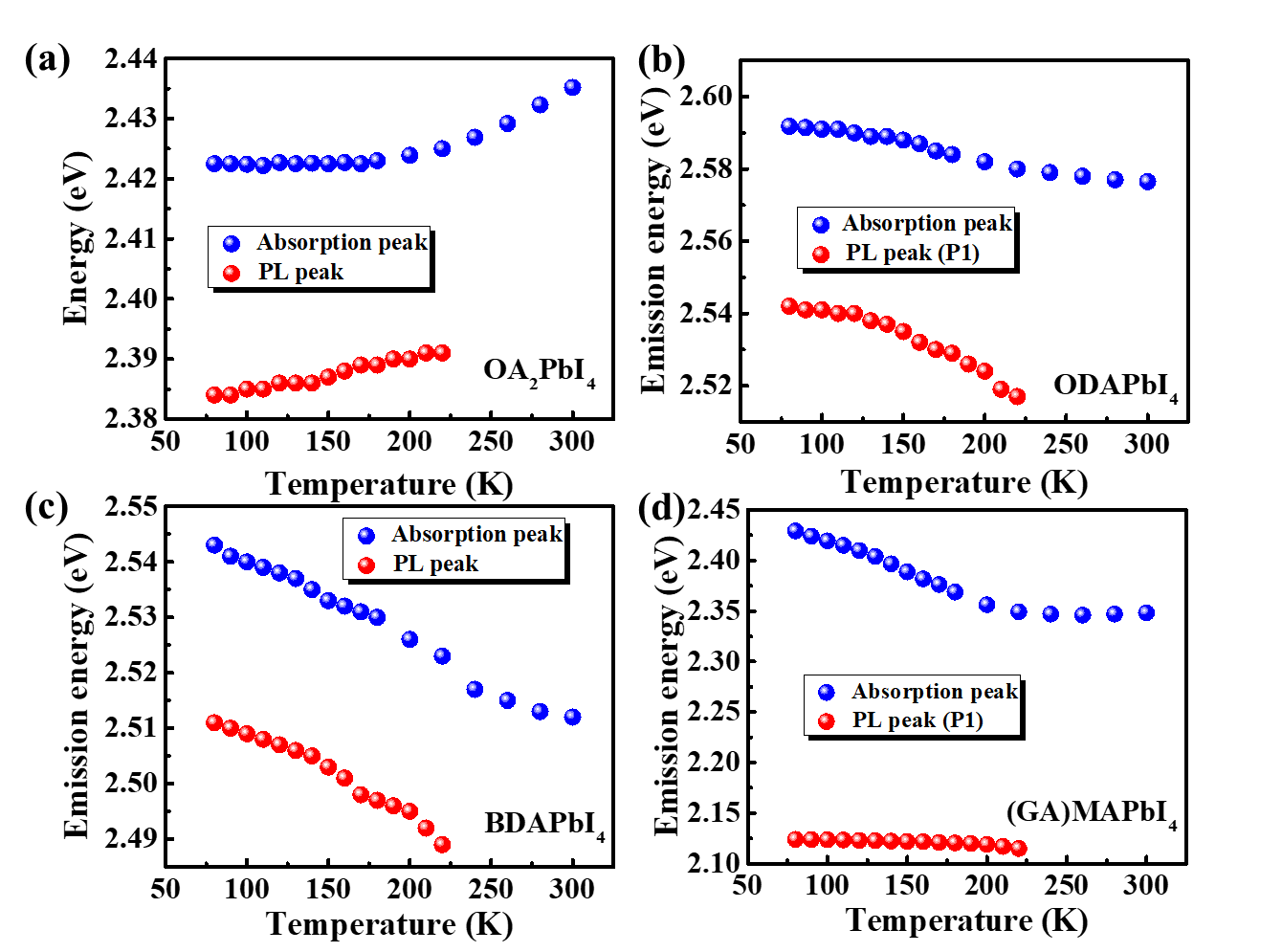
**

**Figure S6.** Comparison of the PL peak energy and exciton absorption peak energy in 2D perovskite. (a) OA_2_PbI_4_. (b) ODAPbI_4_. (c) BDAPbI_4_. (d) (GA)MAPbI_4_.

**
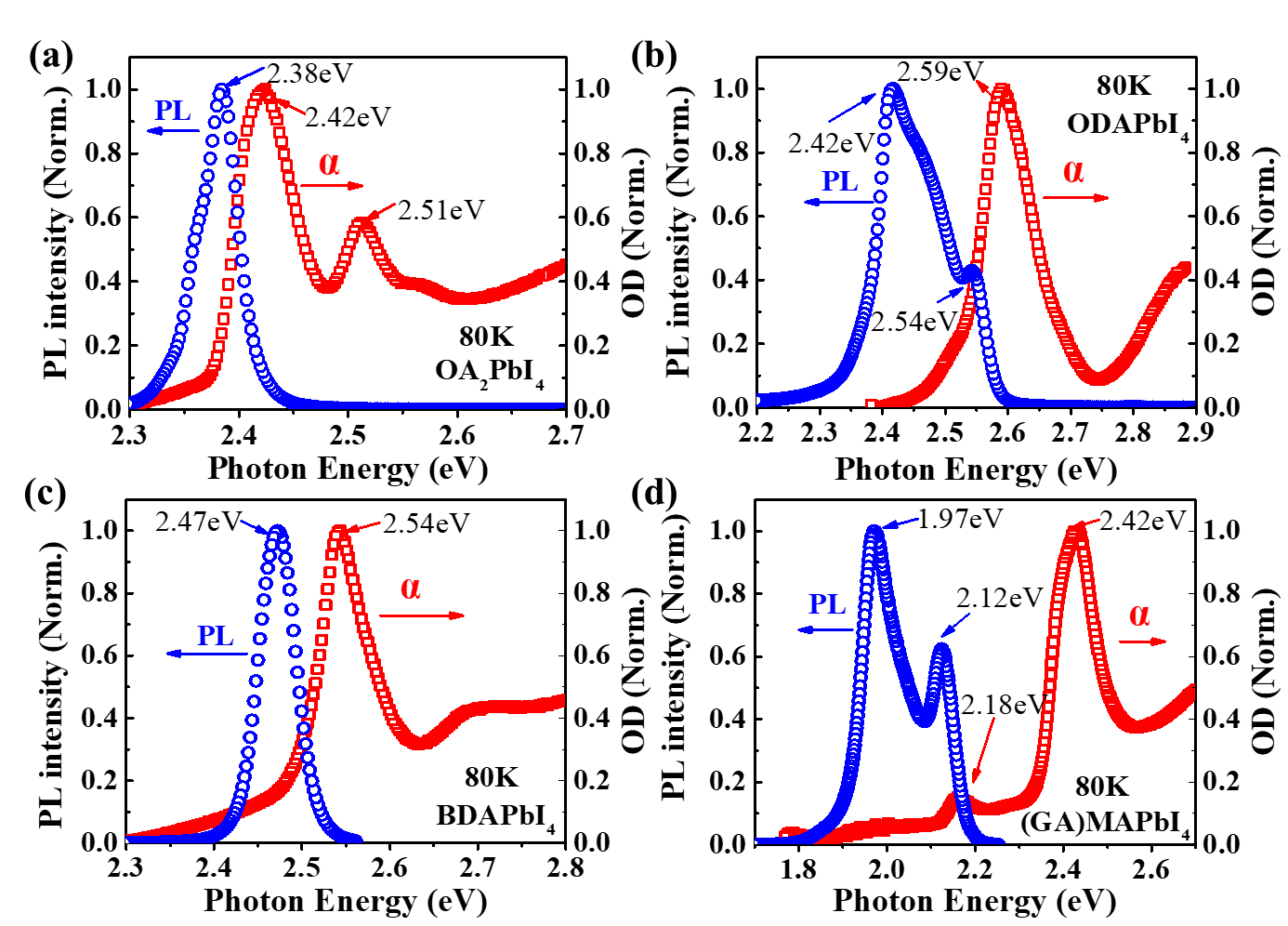
**

**Figure S7.** Comparison of the PL spectra and absorption spectrum in 2D perovskite at 80K. (a) OA_2_PbI_4_. (b) ODAPbI_4_. (c) BDAPbI_4_. (d) (GA)MAPbI_4_.

**
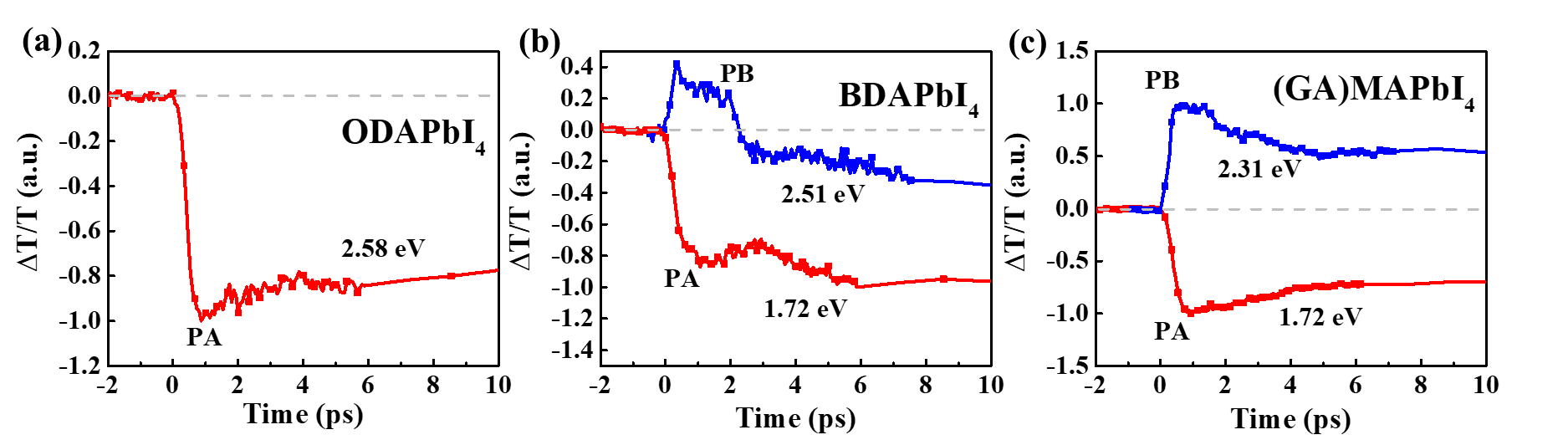
**

**Figure S8.** Transient dynamics for (a) ODAPbI_4_ at 2.58 eV. (b) BDAPbI_4_ at 2.51 eV and 1.72 eV respectively. (c) (GA)MAPbI_4_ at 2.31 eV and 1.72 eV respectively. PA: photoinduced absorption. PB: photoinduced bleaching.

**2D Elliott model for achieving binding energy at room temperature**

Layered hybrid perovskites with *n* = 1 of inorganic slab was generally thought as 2D semiconductors, thus the absorption spectrum can be described as a 2D Wannier-Mott exciton. Following Elliott’s model [6-8], the absorption coefficient can be described following:

Eq. S2

where α_exc_ and α_cont_ are the contributions to absorption from exciton and free carrier continuum respectively. *E_g_* is the band gap energy, *E_b_* = 4**E_0_* is the exciton binding energy, *n* = 1, 2,… is an integer for discrete energy levels for exciton. The spectral width is simulated by hyperbolic secant functions [8, 9], characterized by two parameters Γ_ex_ and Γ_c_ for excitonic and free carrier transitions, respectively. The fitting curves and experimental results are directly compared in following Figure S9. Along with OA_2_PbI_4_, ODAPbI_4_, BDAPbI_4_, and (GA)MAPbI_4_, we also include PEA_2_PbI_4_ in Figure S9a, for which the excion binding energy is known around 200 meV [10, 11].


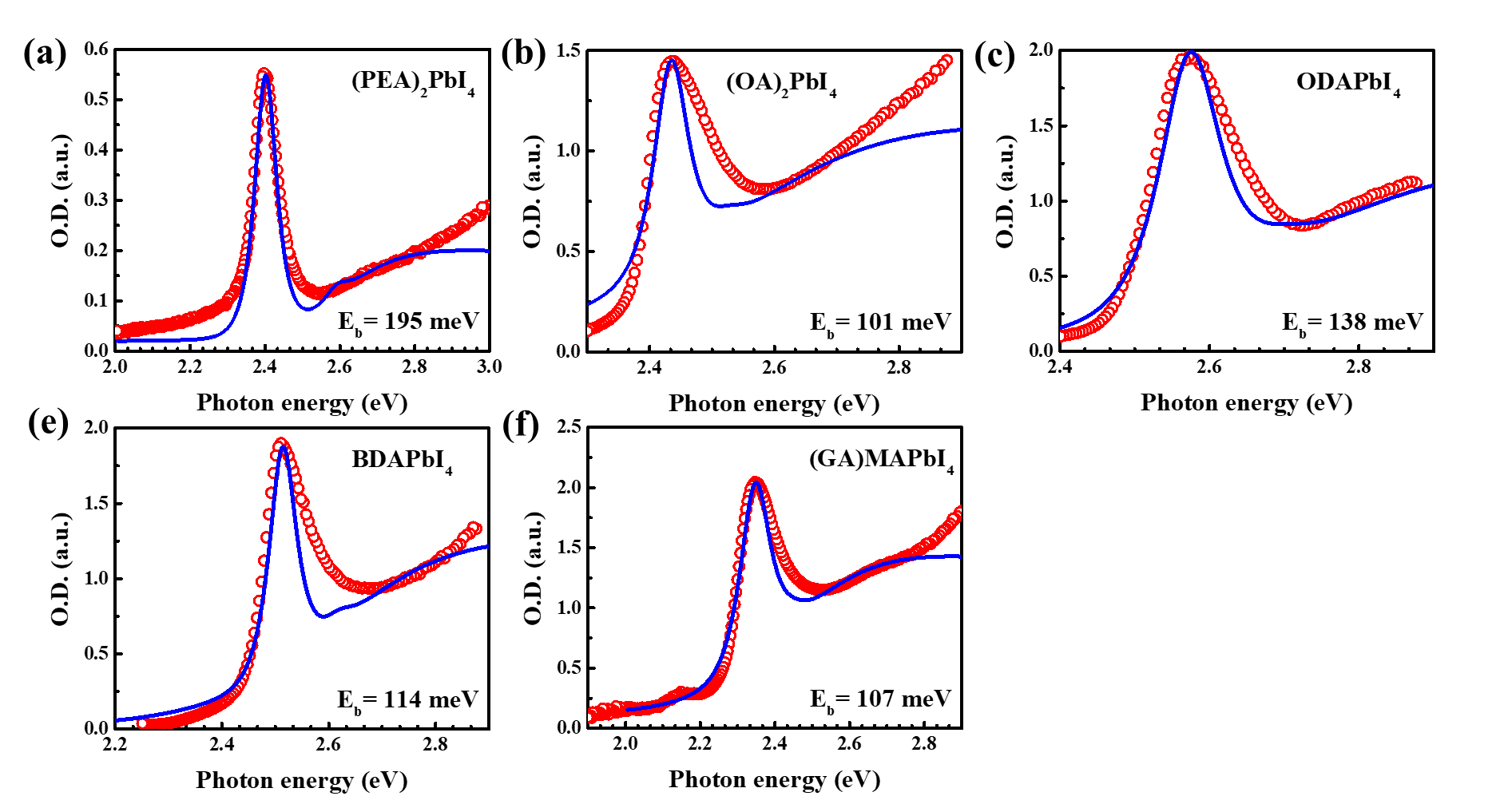


**Figure S9.** Blue line is the fitting curve using 2D Elliott model (Eq. S2 and its discussion), red square are experimental data for absorption spectrum at room temperature.

The fitting parameters of Figure S9 and Eq. S2 are summarized in Table S1. Since we ignore the contribution from phonons [8] and non-parabolicity in the joint dispersion of valance and conduction bands [9], particularly for energy away from bandgap, the fitting results do not fully describe the non-symmetric spectral shape of excitonic transition and absorption of band transition at higher energy. Nevertheless, readily we can conclude that the binding energy of excitons for four 2D perovskite materials at room temperature in current work is on the order of 100 meV.

**Table S1.** The fitting parameters of Eq. S2 and Figure S9 for absorption spectrum using 2D Elliott model. * binding energy of PEA_2_PbI_4_ is same with the reported values [9, 10].

| **Sample** | Γ_ex_ (meV) | Γ_c_ (meV) | E_g_ | E_b_ | **T** |
| --- | --- | --- | --- | --- | --- |
| OA_2_PbI_4_ | 23.9 | 184 | 2.535 | 101 | 300 |
| ODAPbI_4_ | 35.4 | 164 | 2.712 | 138 | 300 |
| BDAPbI_4_ | 22.4 | 153 | 2.628 | 114 | 300 |
| (GA)MAPbI_4_ | 35.2 | 154 | 2.455 | 107 | 300 |
| PEA_2_PbI_4_ ^[7]^ | 26.2 | 91.6 | 2.595 | 195* | 290 |

**References**

[1] T. Sheikh,A. Shinde,S. Mahamuni *et al.*, "Dual excitonic emissions and structural phase transition of octylammonium lead iodide 2D layered perovskite single crystal," *Mater. Res. Express,* vol. 6, no. 12, p. 124002, 2019.

[2] M. Safdari,P. H. Svensson,M. T. Hoang *et al.*, "Layered 2D alkyldiammonium lead iodide perovskites: synthesis, characterization, and use in solar cells," *J. Mater. Chem. A,* vol. 4, no. 40, pp. 15638-15646, 2016.

[3] S. Silver,S. Xun,H. Li *et al.*, "Structural and Electronic Impact of an Asymmetric Organic Ligand in Diammonium Lead Iodide Perovskites," *Adv. Energy Mater.,* vol. 10, no. 14, p. 1903900, 2020.

[4] P. Yan,S. Hu,Y. Zhang *et al.*, "Simple method to synthesize larger n 2D perovskite from (C(NH_2_)_3_)(CH_3_NH_3_)_n_[Pb_n_I_3n+1_] of n = 1 using isopropanol," *Org. Electron.,* vol. 105, p. 106486, 2022.

[5] K. Wei,Z. Xu,R. Chen *et al.*, "Temperature-dependent excitonic photoluminescence excited by two-photon absorption in perovskite CsPbBr_3_ quantum dots," *Opt. Lett.,* vol. 41, no. 16, pp. 3821-3824, 2016.

[6] R. J. Elliott, "Intensity of Optical Absorption by Excitons," *Physical Review,* vol. 108, no. 6, pp. 1384-1389, 1957.

[7] Y. Zhang,R. Wang,Y. Li *et al.*, "Optical properties of two-dimensional perovskite films of (C_6_H_5_C_2_H_4_NH_3_)_2_[PbI_4_] and (C_6_H_5_C_2_H_4_NH_3_)_2_ (CH_3_NH_3_)_2_[Pb_3_I_10_]," *J. Phys. Chem. Lett.,* vol. 10, no. 1, pp. 13-19, 2019.

[8] S. Neutzner,F. Thouin,D. Cortecchia *et al.*, "Exciton-polaron spectral structures in two-dimensional hybrid lead-halide perovskites," *Phys. Rev. Mater.,* vol. 2, no. 6, 2018.

[9] F. Ruf,M. F. Aygüler,N. Giesbrecht *et al.*, "Temperature-dependent studies of exciton binding energy and phase-transition suppression in (Cs,FA,MA)Pb(I,Br)_3_ perovskites," *APL Mater.,* vol. 7, no. 3, p. 031113, 2019.

[10] Yaxin Zhai,Sangita Baniya,Chuang Zhang *et al.*, "Giant Rashba splitting in 2D organic-inorganic halide perovskites measured by transient spectroscopies," *Sci. Adv.,* vol. 3, no. 7, p. e1700704, 2017.

[11] X. Hong, T. Ishihara,A. V. Nurmikko, "Dielectric confinement effect on excitons in PbI4-based layered semiconductors," *Phys Rev B Condens Matter,* vol. 45, no. 12, pp. 6961-6964, 1992.
